# Association of the *APOE* Gene Polymorphism with Depression in White Adults in the WHO “MONICA-Psychosocial” Program

**DOI:** 10.3390/jpm13091306

**Published:** 2023-08-26

**Authors:** Valery Gafarov, Elena Gromova, Elena Shakhtshneider, Igor Gagulin, Almira Gafarova

**Affiliations:** 1 Institute of Internal and Preventive Medicine (IIPM)-Branch of ICG SB RAS, 175/1 Borisa Bogatkova Str., Novosibirsk 630089, Russia; 2 Collaborative Laboratory of Epidemiology of Cardiovascular Diseases (CVD), 175/1 Borisa Bogatkova Str., Novosibirsk 630089, Russia

**Keywords:** *APOE* gene, depression, ε2/ε3/ε4 polymorphism, rs7412, rs429358

## Abstract

The *APOE* gene polymorphism is associated with the risk of the development of several neurological disorders. The aim of the study was to investigate the association of the *APOE* gene polymorphism with depression in the white adult population aged 25–64 years in Novosibirsk (Western Siberia). The third screening of the WHO program “MONICA-psychosocial” was conducted in 1994–1995. In total, 403 men (the average age was 34 ± 0.4 years, the response was 71%) and 531 women (the average age was 35 ± 0.4 years, the response was 72%) of the open population of residents aged 25–64 years of the Oktyabrsky district of Novosibirsk were examined. The “MONICA-MOPSY” psychosocial questionnaire was used to assess depression. A high level of depression was found in 12.8% of the population: in 8.9% of men and in 15.8% of women. The frequencies of *APOE* gene polymorphism genotypes ε2/3, ε2/4, ε3/3, ε3/4, and ε4/4 were 14.9%, 3.1%, 61.6%, 17.5%, and 2.9%, respectively. Carrying the ε3/4 genotype of the *APOE* gene increased the odds of developing major depression by 2.167 times (95% CI 1.100–4.266) compared to carrying the ε3/3 genotype of the *APOE* gene in people without depression (χ^2^ = 5.120 df = 1 *p* = 0.024). Carriers of the ε4 allele were 2.089 times (95% CI 1.160–3.761) more likely to have a high level of depression than those without this allele and no depression (χ^2^ = 6.148 df = 1 *p* = 0.013), and 2.049 times (95% CI 1.117–3.758) more likely to have a moderate level of depression than those without this allele (χ^2^ = 5.470 df = 1 *p* < 0.019). The ε4 allele of the *APOE* gene is associated with a high level of depression.

## 1. Introduction

Depression is a serious mental disorder that affects the health of a person and is characterized by emotional dysfunction [1]. The current view is that depression is a consequence of the interaction between genetic factors and environmental risk factors [2]. One of the risk factors for depression is the *APOE* gene, a number of variants of which have been associated with the development of atherosclerosis and cardiovascular disease, as well as with the development of neurodegenerative disorders and brain pathology [3,4,5,6]. The results of a recent review conducted by J. Alagarsamy et al. showed that population studies revealed the association between APOE polymorphisms with increased risk of coronary and perivascular diseases [3]. In our recent study, we revealed a significant association of the ε2ε4 genotype of the APOE gene with MI (*p* < 0.0001) [4], in agreement with the results of the review performed by J. Alagarsamy et al. [3]. In 2021, A. Serrano-Pozo et al. performed an analysis, confirming that the APOE ε4 allele is a genetic risk factor for sporadic Alzheimer’s disease [5].

The association of the ε3/ε4 genotype of the *APOE* gene with the development of depressive symptoms was first reported in 1996 in a study by Ramachandran et al. [7]. Other studies have shown that there is an association between depression and a number of genotypes of the *APOE* gene [8,9].

Apolipoprotein E (APOE) is the major apolipoprotein of chylomicrons and is necessary for the normal breakdown of triglyceride-rich lipoprotein components. The APOE protein is expressed in many cells, primarily in liver cells, but also in the cells of the brain, spleen, kidneys, gonads, adrenal glands, and macrophages. mRNA of apolipoprotein E has been found in the cerebral cortex and hippocampus [10]. In the brain, APOE is synthesized primarily by astrocytes. The widespread expression of APOE indicates its importance for various processes. Apolipoprotein E acts as a mediator in pathological processes. APOE regulates neuroinflammation and neurotoxicity through direct actions on microglia, astrocytes, and neurons. In general, APOE ε4 promotes pro-inflammatory and neurotoxic processes, while APOE ε2 acts in a neuroprotective manner [9]. Results of these studies can be used for the development of targets for novel therapies to treat depression.

The *APOE* gene is mapped to chromosome 19q13.2. Variants leading to APOE protein isoform formation are rs429358, p.Cys130Arg (ε4) and rs7412, p.Arg176Cys (ε2). According to the frequency of alleles indicated in the Genome Aggregation Database (gnomAD), the frequency of the rs429358 minor allele is 0.14 and the frequency of the rs7412 minor allele is 0.07 in the general gnomAD population [11]. The prevalence rates of the ε2/ε2, ε2/ε3, ε2/ε4, ε3/ε3, ε3/ε4, and ε4/ε4 genotypes in the general population are 0.004, 0.065, 0.009, 0.759, 0.143, and 0.02, respectively [12].

Several studies have shown that certain genotypic polymorphisms in the *APOE* gene coding region are risk factors for depression and other diseases [13,14]. In 2020, Y.P. Huang et al. performed a meta-analysis, showing that, in a longitudinal community-based study of elderly, a positive association was observed between the ε4 allele of *APOE* and depression during 5 years of follow-up. A meta-analysis of nine studies also found that the ε4 allele was positively associated with depression in individuals aged 23 to 83 years [14]. Plasma total cholesterol and low-density lipoprotein concentrations in recovering depressed patients may predict suicide risk during the next depressive episode [15]. Low serum cholesterol concentrations have been associated with the risk of depression [16]. Neuronal loss and structural brain abnormalities, which may be the result of dyslipidemia, are found in many patients with depression [17]. A high frequency of the ε4 allele of the *APOE* gene was found in patients with depression and the ε4 allele was a risk factor for depression [18,19]. Patients with the ε4 allele showed more pronounced symptoms of depression compared to patients without the ε4 allele [20]. The *APOE* genotype may be associated with global brain structure.

Thus, taking into account the above facts, the purpose of our study was to investigate the association of *APOE* gene genotypes with depression in the white adult population aged 25–64 years in Novosibirsk (Western Siberia).

## 2. Materials and Methods

### 2.1. Study Population

The 3rd screening of WHO program “MONICA-psychosocial” was conducted in 1994–1995. In total, 403 men (the average age was 34 ± 0.4 years, the response was 71%) and 531 women (the average age was 35 ± 0.4 years, the response was 72%) of the open population of residents aged 25–64 years of Oktyabrsky district of Novosibirsk were examined. The “MONICA-MOPSY” psychosocial questionnaire was used to assess depression [21]. The study protocol was approved by the Ethics Committee of the IIPM—a branch of the ICG SB RAS, session No. 34 of 23 December 1982. Informed consent was obtained from each subject.

According to the psychosocial questionnaire “MONICA-MOPSY” [21], a high level of depression was determined at 7–12 points, a moderate level of depression at 4–6 points, and the absence of depression at 1–3 points. 

### 2.2. Measures and Clinical Data

The clinical examination program included registration of sociodemographic data; a standard questionnaire on smoking and alcohol consumption; history of chronic diseases; use of medications; the Rose angina questionnaire; anthropometric data (height, body weight, and waist circumference); three-times measurement of blood pressure; spirometry; electrocardiography; and biochemical assays of blood serum (total cholesterol, high-density lipoprotein cholesterol [HDL-C], triglycerides, and fasting glucose). Blood samples were taken from the cubital vein in the morning on an empty stomach and 12 hours after a meal. Blood lipid profiling (total cholesterol, triglycerides, HDL-C, and LDL-C) was performed by enzymatic methods using standard reagents (Biocon Fluitest; Lichtenfels, Germany) on a Labsystem FP-901 biochemistry analyzer (Labsystems Oy, Helsinki, Finland) in 602 subjects. The atherogenicity index was calculated using the formula: IA = (TC − HDL-C)/HDL-C.

### 2.3. Genotyping and Quality Control 

From the total sample, 383 individuals were selected for genotyping using the random number method. Genotyping of the *APOE* gene polymorphism was carried out at the Research Institute of Therapy and Preventive Medicine—branch of ICG SB RAS, Novosibirsk. 

DNA was isolated from blood using the phenol–chloroform extraction method [22]. The quality of the isolated DNA was evaluated using the Agilent 2100 Bioanalyzer capillary electrophoresis system (Agilent Technologies Inc., Santa Clara, CA, USA).

Genomic DNA was amplified by PCR in a standard reaction mixture. It was then hydrolyzed by AspLE I Restriction Enzyme with GCGC recognition site. Visualization of the restriction products was performed by gel electrophoresis in 10% PAAG followed by ethidium bromide staining and computer video scanning of the gel. The physical and clinical examination data were blinded to the laboratory personnel performing the genotyping assays.

### 2.4. Statistical Analyses

SPSS for Windows 23.0 was used for statistical processing of the results. The distribution of the features and their numerical characteristics were the subject of analysis. The analysis of the simple relations between the variables (tables of conjugacy) was carried out. The reliability of the independence of the factors was evaluated according to criterion χ^2^ [23]. The differences in the mean values of quantitative indicators between different genotypes, after standardization by sex, age, and body mass index, were evaluated using the “GLM: univariate model” procedure of the SPSS package of applied statistical programs. Genotype and sex were the fixed variables. Age and body mass index were used as covariates. Continuous variables had a normal distribution. The significance level was set below 0.05.

## 3. Results

Table 1 shows the baseline characteristics of the subjects. Males accounted for 70.8% and females for 29.2%. 

An analysis of the association of rs7412 and rs429358 with blood lipid profile indicators —total cholesterol, HDL-C, LDL-C, and TG, as well as with the atherogenicity index (IA)—was performed (Table 2). The highest mean TS levels were found for the ε4/ε4 genotype in both men and women. 

Both in the general group (*p* = 0.12) and in women (*p* = 0.37), the differences between genotypes in mean LDL-C levels were statistically significant. Maximum LDL-C was found in men, women, and the general group with the ε4/4 genotype.

Higher mean IA values were observed in men and in the general group with the ε4/4 genotype. A statistically significant association was found between the polymorphism of the coding part of the APOE gene and IA level (*p* = 0.027).

### 3.1. Depression Prevalence

In the white adult population aged 25–64 in Novosibirsk (Western Siberia), the prevalence of depression was as follows: a high level of depression was found in 12.8% of the population, in 8.9% of men and in 15.8% of women, and a moderate level in 24.5% of the population (men—21.3%, women—26.9%) (see Table 3).

### 3.2. Allele and Genotype Frequencies of rs429358 and rs7412 (the APOE Gene)

Table 4 shows the frequency of genotypes and alleles of the *APOE* gene in the white adult population aged 25–64 years in Novosibirsk (Western Siberia), for whom the questionnaire “MONICA-MOPSY” was filled out.

The frequencies of *APOE* gene polymorphism genotypes ε2/3, ε2/4, ε3/3, ε3/4, and ε4/4 were 14.9%, 3.1%, 61.6%, 17.5%, and 2.9%, respectively. The frequency of the ε3 allele was 77.8%, of the ε4 allele—13.2% and of the ε2 allele—9.0%.

### 3.3. Prevalence of Depression in Carriers of Various APOE Gene Alleles

Carriers of genotypes containing the ε4 allele (ε2/ε4 + ε3/ε4 + ε4/ε4) were more likely to have high levels of depression (34.5%), whereas carriers of the ε2/ε3 + ε3/ε3 genotypes were free of depression (79.9%) (χ^2^ = 7.559 df = 2 *p* = 0.022) (Table 5). 

Carrying the ε3/ε4 genotype of the *APOE* gene increased the odds of developing high levels of depression by 2.167 (95% CI 1.100–4.266) times compared to the carrier of the ε3/ε3 genotype of the *APOE* gene (χ^2^ = 5.120 df = 1 *p* = 0.024). Carriers of the ε4 allele (ε2/ε4 + ε3/ε4 + ε4/ε4) had high levels of depression 2.089 (95%CI 1.160–3.761) times more often than individuals without this allele (ε2/ε3 + ε3/ε3) without depression (χ^2^ = 6.148 df = 1 *p* = 0.013) and 2.049 (95%CI 1.117–3.758) times more often than those with moderate levels of depression (χ^2^ = 5.470 df = 1 *p* = 0.019). 

Carriers of the ε4 allele were 1.9 times more likely to have high levels of depression (95%CI 1.131–3.193), compared with carriers of the ε3 allele without depression (χ^2^ = 6.001 df = 1 *p* = 0.014), and 1.828 (95% CI 1.071–3.118) more likely compared to those with moderate levels of depression (χ^2^ = 4.985 df = 1 *p* = 0.026) (see Table 6).

## 4. Discussion

In our study, a high level of depression was found in 12.8% of the population: in 8.9% of men and in 15.8% of women. Carrying the ε3/4 genotype of the APOE gene increased the odds of developing major depression by 2.167 times (95%CI 1.100–4.266) compared to carrying the ε3/3 genotype of the APOE gene in people without depression (χ^2^ = 5.120 df = 1 *p* = 0.024). Carriers of the ε4 allele were 2.089 times (95% CI 1.160–3.761) more likely to have a high level of depression than those without this allele and no depression (χ^2^ = 6.148 df = 1 *p* = 0.013), and 2.049 times (95% CI 1.117–3.758) more likely to have a moderate level of depression than those without this allele (χ^2^ = 5.470 df = 1 *p* < 0.0 19). The ε4 allele of the APOE gene is associated with a high level of depression in our study.

When analyzing the association of rs7412 and rs429358 with the parameters of the lipid profile in the white adult population of Novosibirsk (Western Siberia), the maximum values of the average level of TC, LDL-C, and IA were identified for the genotype ε4/ε4 in men and women, which correlates with the data of other studies. According to the results of large global studies using UK Biobank data, the ε4ε4 genotype is associated with higher levels of hypercholesterolemia and coronary heart disease compared to the ε3ε3 genotype [24]. According to another study using UK Biobank data (345,659 Caucasians without a history of coronary artery disease), the worst prognosis of survival without fatal events was in carriers of the ε4 allele [25].

The most well-studied function of the apoE protein is its role as a ligand responsible for the removal of triglyceride- and cholesterol-rich lipoproteins from circulating plasma by binding with high affinity to the LDL receptor on the surface of hepatocytes [26,27].

According to the literature, carrying the ε4 allele increases the risk of neurological disorders by increasing amyloid accumulation, promoting tau hyperphosphorylation, and exacerbating neuroinflammation in the brain [28,29,30,31]. J. Iannucci et al. obtained evidence that support a pro-inflammatory and neurotoxic role for apolipoprotein ε4, along with a neuroprotective one for apolipoprotein ε2 [31]. Higher expression of *APOE* has also been found in glial cells of the central nervous system, suggesting an involvement of *APOE* in neuroinflammation [32]. 

A meta-analysis of 20 studies showed an association between a polymorphism in the *APOE* gene and a predisposition to depression. The ε4 allele has been associated with an increased risk of depression in people with late-life depression [33].

In the white adult population of Novosibirsk (Western Siberia), depression among 25–64 year olds occurred in 12.8% of the population, including 8.9% of men and 15.8% of women, which is a global trend [34]. With the development of progress, the incidence of depression in the world is increasing year by year, affecting thoughts, mood, and physical health. Depression completely changes people’s understanding of the world and interpersonal relationships, and contributes to an increase in the number of suicides [34]. 

In the white adult population aged 25–64 in Novosibirsk (Western Siberia), for whom the questionnaire “MONICA-MOPSY” was filled out, the homozygous genotype ε3/ε3 (61.6%) of the *APOE* gene, heterozygous genotypes ε3/ε4 and ε2/ε3 were found in 17.5% and 14.9% of the population, respectively. The ε3 allele was observed in 77.8% of individuals, the ε4 allele in 13.2% and the ε2 allele in 9%. Our data are in agreement with the results of researchers from around the world [12,14,15] as well as from domestic researchers [4,35]. For example, 70.4% had the ε3/ε3 genotype, 9% had the ε3/ε4 genotype, and 6% had the ε2/ε3 genotype, according to I.B. Zueva et al., 2012 [35].

In the white adult population aged 25–64 years in Novosibirsk (Western Siberia), high levels of depression were observed more frequently (19%) among those with the ε4 allele genotype. Carrying the ε3/ε4 genotype of the *APOE* gene more than doubled the odds of developing major depression compared to carriers of the ε3/ε3 genotype. Individuals with the e4 allele present in their genotype (ε2/ε4 + ε3/ε4 + ε4/ε4 genotypes) had a high level of depression two times more often than those with the ε2/ε3 + ε3/ε3 genotypes, who either did not have depression or the level of depression was moderate. High levels of depression were observed 1.9 times more frequently in carriers of the ε4 allele than in those with no depression and 1.28 times more frequently than in those with moderate levels of depression. Our data are consistent with both the results of domestic researchers, who concluded that carrying the ε4 allele of the *APOE* gene is an unfavorable factor contributing to the development of depression [35], and the results of a large-scale meta-analysis conducted by W.W. Wang, et al. 2019 [36]. The meta-analysis was a review of 398 sources of information on the association between the ε4 allele of the *APOE* gene and depression. The authors used genetic models such as the allelic model (ε4 vs. ε2 + ε3), the dominant model (ε4/ε4 + ε4/ε3 + ε4/ε2 vs. ε2/ε3 + ε2/ε2 + ε3/ε3), and the recessive model (ε4/ε4 vs. ε2/ε2 + ε2/ε3 + ε3/ε3 + ε2/ε4 + ε3/ε4). The ε4 allele of the *APOE* gene was statistically significantly associated with depression: OR = 1.360, 95% CI 1.110–1.660, *p* = 0.003, in the dominant model: OR = 1.340, 95% CI 1.060–1.680, *p* = 0.001 [36]. Possible pathophysiological mechanisms for the development of depression in carriers of the ε4 allele of the *APOE* gene include impaired regulation of lipid metabolism, as well as immune–inflammatory imbalance and increased oxidative stress [37]. The ε3 allele is associated with fewer depressive symptoms, and depressive symptoms are negatively correlated with LDL-C [38]. According to the literature data, the combination of the ε4 allele carrier and modifiable risk factors conveys a stronger association with incident cognitive impairment, not dementia than either type of risk factor alone [39].

Limitations: We analyzed only rs429358 and rs7412 (the *APOE* gene) and therefore could not exclude the influence of other factors that may affect observational studies. Biochemical blood assays in our patients did not include remnant cholesterol.

## 5. Conclusions

High levels of depression are observed in 12.8% of the white adult population aged 25–64 in Novosibirsk (Western Siberia). The ε4 allele of the *APOE* gene is associated with a high level of depression. The study of genetic risk factors for depression in different populations is important not only for the analysis of disease outcomes but also for the formation of primary prevention groups. 

## Figures and Tables

**Table 1:** Baseline characteristics of the subjects.

	Males	Females	Total
Number of subjects	426	176	602
Age, years	45.0 ± 9.5	46.2 ± 11.6	45.3 ± 10.1
TC, mg/dL	206.44 ± 41.8	204.8 ± 49.1	206.3 ± 41.6
HDL-C, mg/dL	54.6 ± 16.9	58.0 ± 13.0	54.3 ± 17.2
LDL-C, mg/dL	129.3 ± 40.7	127.3 ± 45.6	128.7 ± 42.2
TGs, mg/dL	114.5 ± 70.0	99.3 ± 48.3	114.4 ± 70.0
Index of atherogenicity	3.1 ± 1.4	2.7 ± 1.2	3.0 ± 1.4
Body mass index, kg/m^2^	25.7 ± 4.1	28.6 ± 5.5	26.5 ± 4.7
Systolic blood pressure, mmHg	132.2 ± 20.2	136.5 ± 26.5	133.5 ± 22.3
Diastolic blood pressure, mmHg	85.8 ± 12.0	86.5 ± 13.1	86.0 ± 12.5

Continuous variables are presented as mean ± standard deviation. TGs, triglycerides; TC, total cholesterol; HDL-C, high-density lipoprotein cholesterol; LDL-C, low-density lipoprotein cholesterol.

**Table 2:** Associations of rs429358 and rs7412 genotypes with blood lipid profile parameters (n = 602).

Sex	Genotype	TC, mg/dL	HDL-C, mg/dL	LDL-C, mg/dL	TGs, mg/dL	Index of Atherogenicity
Males	ε2/ε4	202.3 ± 41.3	47.3 ± 12.4	129.0 ± 42.4	129.6 ± 54.9	3.6 ± 1.6
	ε2/ε3	203.1 ± 39.8	58.3 ± 18.7	118.7 ± 37.1	126.6 ± 98.3	2.7 ± 1.3
	ε3/ε3	206.7 ± 43.4	55.3 ± 16.8	129.7 ± 42.7	111.0 ± 67.4	3.0 ± 1.6
	ε3/ε4	204.3 ± 35.1	50.4 ± 13.9	131.0 ± 32.9	114.9 ± 60.7	3.3 ± 1.4
	ε4/ε4	235.7 ± 44.1	52.9 ± 24.9	156.9 ± 40.7	129.3 ± 47.6	4.1 ± 1.8
*p*		0.034 *	0.035 *	0.123	0.426	0.027 *
Females	ε2/ε4	-	-	-	-	-
	ε2/ε3	192.5 ± 55.7	58.4 ± 16.1	110.4 ± 54.2	118.9 ± 66.6	2.3 ± 1.7
	ε3/ε3	210.2 ± 47.8	58.2 ± 12.7	133.0 ± 44.7	97.8 ± 41.9	2.8 ± 1.1
	ε3/ε4	192.2 ± 47.6	56.9 ± 12.1	117.0 ± 40.6	91.8 ± 53.2	2.5 ± 0.9
	ε4/ε4	257.0 ± 49.2	79.0 ± 13.0	162.8 ± 45.6	76.0 ± 48.3	2.3 ± 1.2
*p*		0.93	0.335	0.037 *	0.460	0.488
Total	ε2/ε4	202.3 ± 41.3	47.3 ± 12.4	129.0 ± 42.4	129.5 ± 54.9	3.5 ± 1.6
	ε2/ε3	200.0 ± 44.8	58.3 ± 17.8	116.2 ± 42.7	124.4 ± 89.9	2.7 ± 1.4
	ε3/ε3	207.7 ± 44.6	56.1 ± 15.8	130.6 ± 43.3	107.3 ± 61.6	3.0 ± 1.4
	ε3/ε4	199.95 ± 40.3	52.7 ± 13.6	125.9 ± 36.3	106.5 ± 58.9	3.0 ± 1.3
	ε4/ε4	237.6 ± 42.3	55.3 ± 24.9	157.5 ± 32.1	124.5 ± 64.9	3.9 ± 1.8
*p*		0.075	0.051	0.012 *	0.351	0.027 *

The data are presented as mean ± standard deviation. * Statistically significant.

**Table 3:** Prevalence of depression in the white adult population aged 25–64 in Novosibirsk (Western Siberia).

Depression	Total		Males		Females	
	n	%	n	%	n	%
Not found	585	62.6	281	69.7	304	57.3
Moderate	229	24.5	86	21.3	143	26.9
Severe	120	12.8	36	8.9	84	15.8
Total	934	100.0	403	100.0	531	100.0

**Table 4:** The frequency of genotypes and alleles of the *APOE* gene in the white adult population aged 25–64 years in Novosibirsk (Western Siberia), n = 383.

**Genotypes**	**n**	**%**
ε2/ε3	57	14.9
ε2/ε4	12	3.1
ε3/ε3	236	61.6
ε3/ε4	67	17.5
ε4/ε4	11	2.9
**Alleles**	**n**	**%**
ε2	69	9.0
ε3	596	77.8
ε4	101	13.2

**Table 5:** Depression and *APOE* gene genotypes in the white adult population aged 25–64 from Novosibirsk (Western Siberia).

	No Depression		Moderate Levels of Depression		High Levels of Depression	
Genotypes	n	%	n	%	n	%
ε2/ε3	23	14.5	21	15.3	13	15
ε2/ε4	4	2.5	3	2.2	5	5.7
ε3/ε3	104	65.4	88	64.2	44	50.6
ε3/ε4	24	15.1	21	15.3	22	25.3
ε4/ε4	4	2.5	4	3	3	3.4
	χ^2^ = 8.848 df = 8 *p* > 0.05					
ε2/ε3 + ε3/ε3 model	127	79.9	109	79.6	57	65.5
ε2/ε4 + ε3/ε4 + ε4/ε4 model	32	20.1	28	20.4	30	34.5
	χ^2^ = 7.559 df = 2 *p* = 0.022					
alleles	n	%	n	%	n	%
ε2	27	8.5	24	8.7	18	10.3
ε3	255	80.2	218	79.6	123	70.7
ε4	36	11.3	32	11.7	33	19.0
	χ^2^ = 7.651 df = 4 *p* > 0.05					

**Table 6:** Comparative analysis of the incidence of depression in the white adult population aged 25–64 years in Novosibirsk (Western Siberia) with different genotypes of the polymorphism of the coding part of the *APOE* gene.

Genotypes	Depression				Pearson’s χ^2^/ Fisher’s Exact Test/ Odds Ratio/ 95% CI OR
	No		High level		
	n	%	n	%	
Carriers of the ε3/ε3 genotype	104	81.2	44	66.7	5.120 df = 1 *p* = 0.024 0.032 2.167 1.100–4.266
Carriers of the ε3/ε4 genotype	24	18.8	22	33.3	
	No		High level		
	n	%	n	%	
Carriers of the ε2/2 + ε2/3 + ε3/3 genotypes	127	79.9	57	65.5	6.148 df = 1 *p* = 0.013 0.021 2.089 1.160–3.761
Carriers of the ε2/ε4 + ε3/ε4 + ε4/ε4 genotypes	32	20.1	30	34.5	
	Moderate levels		High level		
	n	%	n	%	
Carriers of the ε2/2 + ε2/3 + ε3/3 genotypes	109	79.6	57	65.5	5.470 df = 1 *p* = 0.019 0.028 2.049 1.117–3.758
Carriers of the ε2/ε4 + ε3/ε4 + ε4/ε4 genotypes	28	20.4	30	34.5	
	No		High level		
	n	%	n	%	
Carriers of the ε3 allele	255	87.6	123	78.8	6.001 df = 1 *p* = 0.014 0.019 1.900 1.131–3.193
Carriers of the ε4 allele	36	12.4	33	21.2	
	Moderate levels		High level		
	n	%	n	%	
Carriers of the ε3 allele	218	87.2	123	78.8	4.985 df = 1 *p* = 0.026 0.036 1.828 1.071–3.118
Carriers of the ε4 allele	32	12.8	33	21.2	

## Data Availability

The data presented in this study are available on request from the corresponding author. The data are not publicly available due to privacy concerns.

## References

[1] Yu S., Holsboer F., Almeida O.F. (2008). Neuronal actions of glucocorticoids: Focus on depression. J. Steroid Biochem. Mol. Biol..

[2] Xu J., He K., Zhang K., Yang C., Nie L., Dan D., Liu J., Zhang C.E., Yang X. (2021). Low-Dose Copper Exposure Exacerbates Depression-Like Behavior in ApoE4 Transgenic Mice. Oxid. Med. Cell Longev..

[3] Alagarsamy J., Jaeschke A., Hui D.Y. (2022). Apolipoprotein E in Cardiometabolic and Neurological Health and Diseases. Int. J. Mol. Sci..

[4] Semaev S., Shakhtshneider E., Shcherbakova L., Orlov P., Ivanoshchuk D., Malyutina S., Gafarov V., Voevoda M., Ragino Y. (2023). Association of Common Variants of APOE, CETP, and the 9p21.3 Chromosomal Region with the Risk of Myocardial Infarction: A Prospective Study. Int. J. Mol. Sci..

[5] Serrano-Pozo A., Das S., Hyman B.T. (2021). APOE and Alzheimer’s disease: Advances in genetics, pathophysiology, and therapeutic approaches. Lancet Neurol..

[6] Goltermann J., Redlich R., Dohm K., Zaremba D., Repple J., Kaehler C., Grotegerd D., Förster K., Meinert S., Enneking V. (2019). Apolipoprotein E Homozygous ε4 Allele Status: A Deteriorating Effect on Visuospatial Working Memory and Global Brain Structure. Front. Neurol..

[7] Ramachandran G., Marder K., Tang M., Schofield P.W., Chun M.R., Devanand D.P., Stern Y., Mayeux R. (1996). A preliminary study of apolipoprotein E genotype and psychiatric manifestations of Alzheimer’s disease. Neurology.

[8] Krishnan K.R., Tupler L.A., Ritchie J.C., McDonald W.M., Knight D.L., Nemeroff C.B., Carroll B.J. (1996). Apolipoprotein E-epsilon 4 frequency in geriatric depression. Biol. Psychiatry.

[9] Yen Y.C., Rebok G.W., Gallo J.J., Yang M.J., Lung F.W., Shih C.H. (2007). ApoE4 allele is associated with late-life depression: A population-based study. Am. J. Geriatr. Psychiatry.

[10] Marias A.D. (2019). Apolipoprotein E in lipoprotein metabolism, health and cardiovascular disease. Pathology.

[11] Karczewski K.J., Francioli L.C., Tiao G., Cummings B.B., Alföldi J., Wang Q., Collins R.L., Laricchia K.M., Ganna A., Birnbaum D.P. (2020). The mutational constraint spectrum quantified from variation in 141,456 humans. Nature.

[12] Khalil Y.A., Rabès J.P., Boileau C., Varret M. (2021). *APOE* gene variants in primary dyslipidemia. Atherosclerosis.

[13] Liu Y.L., Zhang H.M., Pan H.M., Bao Y.H., Xue J., Wang T.C., Dong X.C., Li X.L., Bao H.G. (2016). The relationship between apolipoprotein E gene ε2/ε3/ε4 polymorphism and breast cancer risk: A systematic review and meta-analysis. Onco Targets Ther..

[14] Huang Y.P., Xue J.J., Li C., Chen X., Fu H.J., Fei T., Bi P.X. (2020). Depression and APOEε4 Status in Individuals with Subjective Cognitive Decline: A Meta-Analysis. Psychiatry Investig..

[15] Maes M., Smith R., Christophe A., Vandoolaeghe E., Van Gastel A., Neels H., Demedts P., Wauters A., Meltzer H.Y. (1997). Lower serum high-density lipoprotein cholesterol (HDL-C) in major depression and in depressed men with serious suicidal attempts: Relationship with immune-inflammatory markers. Acta Psychiatry Scand..

[16] Partonen T., Haukka J., Virtamo J., Taylor P.R., Lönnqvist J. (1999). Association of low serum total cholesterol with major depression and suicide. Br. J. Psychiatry.

[17] Butters M.A., Sweet R.A., Mulsant B.H., Ilyas Kamboh M., Pollock B.G., Begley A.E., Reynolds C.F., DeKosky S.T. (2003). APOE is associated with age-of-onset, but not cognitive functioning, in late-life depression. Int. J. Geriatr. Psychiatry.

[18] Löwe L.C., Gaser C., Franke K., Alzheimer’s Disease Neuroimaging Initiative (2016). The Effect of the APOE Genotype on Individual BrainAGE in Normal Aging, Mild Cognitive Impairment, and Alzheimer’s Disease. PLoS ONE..

[19] Choe Y.M., Suh G.H., Lee B.C., Choi I.G., Lee J.H., Kim H.S., Hwang J., Kim J.W. (2022). Brain Amyloid Index as a Probable Marker Bridging Between Subjective Memory Complaint and Objective Cognitive Performance. Front. Neurosci..

[20] Piers R.J., Liu Y., Ang T.F.A., Tao Q., Au R., Qiu W.Q. (2021). Association Between Elevated Depressive Symptoms and Cognitive Function Moderated by APOE4 Status: Framingham Offspring Study. J. Alzheimers Dis..

[21] World Health Organization (1988). MONICA Psychosocial Optional Study. Suggested Measurement Instruments.

[22] Sambrook J., Russel D.W. (2006). Purification of nucleic acids by extraction with phenol:chloroform. CSH Protoc..

[23] Bühl A., Zöfel P. (2000). SPSS Version 10. Einführung in die moderne Datenanalyse unter Windows [SPSS version 10. Introduction to modern data analysis under Windows].

[24] Lumsden A.L., Mulugeta A., Zhou A., Hyppönen E. (2020). Apolipoprotein E (APOE) genotype-associated disease risks: A phenome-wide, registry-based, case-control study utilising the UK Biobank. EBioMedicine.

[25] Bos M.M., Noordam R., Blauw G.J., Slagboom P.E., Rensen P.C.N., Heemst D. (2019). The ApoE ε4 Isoform: Can the Risk of Diseases be Reduced by Environmental Factors?. J. Gerontol. Ser. A.

[26] Mahley R.W., Weisgraber K.H., Huang Y. (2009). Apolipoprotein E: Structure determines function, from atherosclerosis to Alzheimer’s disease to AIDS. J. Lipid Res..

[27] Getz G.S., Reardon C.A. (2009). Apoprotein E as a lipid transport and signaling protein in the blood, liver, and artery wall. J. Lipid Res..

[28] Tachibana M., Holm M.-L., Liu C.-C., Shinohara M., Aikawa T., Oue H., Yamazaki Y., Martens Y.A., Murray M.E., Sullivan P.M. (2019). APOE4-mediated amyloid-β pathology depends on its neuronal receptor LRP1. J. Clin. Investig..

[29] Vasilevskaya A., Taghdiri F., Burke C., Tarazi A., Naeimi S.A., Khodadadi M., Goswami R., Sato C., Grinberg M., Moreno D. (2020). Interaction of APOE4 alleles and PET tau imaging in former contact sport athletes. NeuroImage Clin..

[30] Yu Y., Yang M., Zhuang X., Pan J., Zhao Y., Yu Y. (2022). Effects of toxic apolipoprotein E fragments on Tau phosphorylation and cognitive impairment in neonatal mice under sevoflurane anesthesia. Brain Behav..

[31] Iannucci J., Sen A., Grammas P. (2021). Isoform-specific effects of apolipoprotein E on markers of inflammation and toxicity in brain glia and neuronal cells in vitro. Curr. Issues Mol. Biol..

[32] Mahley R.W. (2016). Central nervous system lipoproteins: ApoE and regulation of cholesterol metabolism. Arterioscler. Thromb. Vasc. Biol..

[33] Feng F., Lu S.S., Hu C.Y., Gong F.F., Qian Z.Z., Yang H.Y., Wu Y.L., Zhao Y.Y., Bi P., Sun Y.H. (2015). Association between apolipoprotein E gene polymorphism and depression. J. Clin. Neurosci..

[34] GBD 2017 Disease and Injury Incidence and Prevalence Collaborators (2018). Global, regional, and national incidence, prevalence, and years lived with disability for 354 diseases and injuries for 195 countries and territories, 1990–2017: A systematic analysis for the Global Burden of Disease Study 2017. Lancet.

[35] Zueva I.B., Ulitina A.S., Ghorab D.N., Moskalenko M.V., Dubina M.V. (2012). Apo E gene polymorphism in patients with metabolic syndrome and cognitive disorders. Arter. Gipertenz..

[36] Wang W.W., Liu X.L., Ruan Y., Wang L., Bao T.H. (2019). Depression was associated with apolipoprotein E ε4 allele polymorphism: A meta-analysis. Iran. J. Basic. Med. Sci..

[37] Zhao F., Yue Y., Jiang H., Yuan Y. (2019). Shared genetic risk factors for depression and stroke. Prog. Neuropsychopharmacol. Biol. Psychiatry..

[38] Li W., Ban C., Yue L., Sun L., Li X., Xiao S. (2020). Homozygosity in the APOE 3 Polymorphism Is Associated With Less Depression and Higher Serum Low-Density Lipoprotein in Chinese Elderly Schizophrenics. Front. Endocrinol..

[39] Kolli A., Zhou Y., Chung G., Ware E.B., Langa K.M., Ehrlich J.R. (2022). Interactions between the apolipoprotein E4 gene and modifiable risk factors for cognitive impairment: A nationally representative panel study. BMC Geriatr..

